# Single-cell RNA sequencing and large-panel NGS analysis reveal transcriptional heterogeneity and genomic characteristics of double primary lung cancer and thyroid cancer

**DOI:** 10.1016/j.gendis.2025.101889

**Published:** 2025-10-22

**Authors:** Erteng Jia, Lixia Zhang, Yong Ge, Li Li, He Zhang, Hao Zhang

**Affiliations:** a Thoracic Surgery Laboratory, The First College of Clinical Medicine, Xuzhou Medical University, Xuzhou, Jiangsu 221006, China; b Department of Thoracic Surgery, Affiliated Hospital of Xuzhou Medical University, Xuzhou, Jiangsu 221006, China; c The College of Life Sciences, Xuzhou Medical University, Jiangsu 221006, China

Double primary malignant tumors refer to the presence of two independent primary malignancies in the same or different organs.^1^ In recent years, the detection rate of double primary malignant tumors has significantly increased, of which double primary cancers related to lung cancer account for 10%–15%.^2^ This is mainly due to the increase in the incidence rate of lung cancer, the long-term side effects of chemotherapy or radiation therapy, and continuous innovations in cancer-related detection techniques.^3^^,^^4^ However, research on double primary malignant tumors (such as lung cancer and thyroid cancer: DPLT) involving lung cancer is still limited. The lack of experimental evidence and clinical data related to double primary tumors, especially the unique genomic and transcriptome characteristics, poses difficulties for the prevention and treatment of double primary tumors.^5^ In this study, we used single-cell RNA sequencing and large-panel sequencing to investigate multiomics changes during DPLT progression and revealed their gene mutation characteristics and tumor microenvironment heterogeneity, thereby providing novel insights for clinical detection and therapeutic strategies in DPLT patients.

Deep sequencing was performed on lung cancer tissues from 18 DPLT (double primary lung cancer, DPLC) and 15 single primary lung cancer (SPLC) patients using large-panel sequencing (Fig. S1A). We found that SPLC patients mainly had TP53 (60%), EGFR (53.3%), and PIK3CA (20%) mutations (Fig. S1B), while DPLC patients showed higher EGFR (33.3%) and ERBB2 (22.2%) mutations (Fig. 1A). This indicates genomic heterogeneity of lung cancer tissues between the two groups of patients. Interestingly, more gene fusions were detected in the SPLC group, while no gene fusions were detected in the DPLC group (Table S1). The results also showed that the average age of the DPLC group was significantly lower than the SPLC group (Fig. S1C). Tumor mutation burden analysis showed that DPLC patients had reduced tumor mutation burden (Fig. S1D). Current research showed that as age increased, somatic mutations were significant and typically tissue-specific, which may explain the increased tumor mutation burden in SPLC patients.

**Figure 1:**
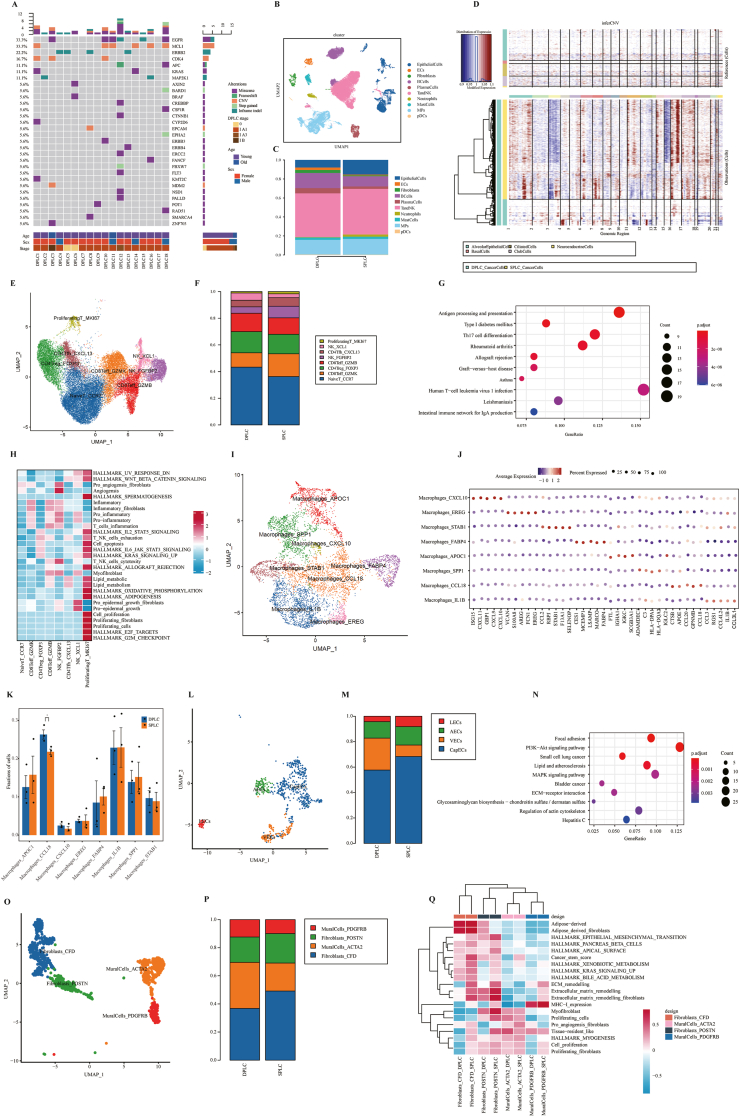
Single-cell transcriptome atlas and large panel sequencing analysis. **(A)** Mutational profile of double primary lung cancer (DPLC) patients. **(B)** Uniform manifold approximation and projection (UMAP) plot of high-quality single cells for cell type identification. **(C)** Proportions of the identified ten major cell types in DPLC and single primary lung cancer (SPLC) patients. **(D)** Copy number variation analysis of DPLC and SPLC patients. **(E)** UMAP plot of 30,782 T cells revealed eight cell subsets. **(F)** Proportions of the identified eight cell subsets in DPLC and SPLC patients. **(G)** Kyoto Encyclopedia of Genes and Genomes (KEGG) enrichment analysis of up-regulated differentially expressed genes in CD8Teff_GZMK cell clusters. **(H)** Single-cell gene set variation analysis (scGSVA) revealed the functions of different cell subsets. **(I)** UMAP plot of macrophage cells revealed eight clusters. **(J)** The dot plot showed the average expression of signature genes of macrophage cell subsets. **(K)** Proportions of the identified eight cell subsets in DPLC and SPLC patients. **(L)** UMAP plot of 1005 endothelial cells revealed four clusters. **(M)** Proportions of the identified four cell subsets in DPLC and SPLC patients. **(N)** KEGG enrichment analysis of up-regulated differentially expressed genes in valvular endothelial cell (VEC) clusters. **(O)** UMAP plot of fibroblasts and mural cells revealed four clusters. **(P)** Proportions of the identified four cell subsets in DPLC and SPLC patients. **(Q)** GSVA of the functions of different cell subsets.

We collected lung cancer samples from three DPLT patients and three SPLC patients for single-cell RNA sequencing (Fig. S1A). 71,448 cells (24,030 for DPLT and 47,418 for SPLC) were divided into ten different subsets (Fig. 1B; Table S2). The proportions of epithelial cells, mononuclear phagocytes, T/natural killer cells, and neutrophils in the DPLC group were significantly lower than in the SPLC group, while the proportions of endothelial cells, plasma cells, and fibroblasts were higher in the DPLC group than in the SPLC group (Fig. 1C). In addition, cancer cells in DPLC had lower copy number variation, revealing high heterogeneity in copy number variation between the two groups (Fig. 1D; Fig. S2A).

To investigate the potential role of T/natural killer cells in DPLT development, we identified eight cell subsets using canonical marker genes (Fig. 1E). Compared with SPLC, the abundance of CD8Teff_GZMK cell clusters in DPLC was significantly decreased (Fig. 1F), and differentially expressed genes mainly involved MHC II protein complex-related biological functions, as well as antigen processing and presentation pathways (Fig. 1G; Fig. S2B). The abundance of CD8Teff_GZMK cell subsets was age-related and could promote an inflammatory phenotype by increasing the secretion of granzyme K (GZMK). This indicates that DPLC patients have a better prognosis than SPLC patients. Single-cell gene set variation analysis (GSVA) found that CD8Teff_GZMK cell subsets were mainly involved in pro-inflammation and inflammatory fibroblasts (Fig. 1H). In addition, killer cell lectin-like receptor K1 (KLRK1) was specifically highly expressed in the CD8Teff_GZMK cell subsets of DPLC patients (Fig. S2C). KLRK1, as a key activating receptor for natural killer cells, directly kills malignant cells in the early stages of tumors by triggering innate immune surveillance mechanisms. Taken together, the CD8Teff_GZMK cell clusters play an important role in the anti-tumor immune response of DPLT patients.

To further investigate macrophage heterogeneity, macrophages were divided into eight cell subpopulations (Fig. 1I and J). The cell subsets showed low expression of pro-inflammatory cytokines (CCL20, CCL17, CXCL8, CCL3, CCL4, and IL1B) in DPLC compared with SPLC (Fig. S2D). The abundance of Macrophages_CCL18 cell subsets was significantly increased in DPLC (Fig. 1K). The enrichment results from gene ontology (GO) analysis and gene set enrichment analysis (GSEA) suggest that the down-regulated genes in DPLC mainly participate in regulating the apoptotic signaling pathway, tumor necrosis factor alpha (TNFα) signaling via nuclear factor kappa B (NF-κB), and pro-inflammation (Fig. S2E and S2F). Notably, the expression levels of pro-inflammatory chemokines CXCL8, CCL20, and CCL17 were significantly reduced in DPLC patients in Macrophages_CCL18 cell clusters (Fig. S2D). The interferon gamma inducible protein 30 (IFI30) gene was highly expressed in the DPLC group (Fig. S2D). It was associated with activated immune response, which may be involved in macrophage activation, T cell-related signal transduction, and antigen-presenting cell processes. Therefore, macrophages_CCL18 cell clusters can inhibit tumor activity in DPLC patients.

In addition, the Kyoto Encyclopedia of Genes and Genomes (KEGG) analysis results showed that up-regulated differentially expressed genes in endothelial cells, epithelial cells, and fibroblasts were mainly involved in endocrine-related pathways such as the gonadotropin hormone-releasing hormone (GnRH) signaling pathway and insulin signaling pathway (Table S3). In contrast, down-regulated differentially expressed genes were mainly involved in autoimmune thyroid disease (Table S4). We found that the proportion of valvular endothelial cell subsets in DPLC was significantly higher than that in the SPLC group (Fig. 1L and M), and the up-regulated differentially expressed genes were mainly involved in the phosphatidylinositol 3-kinase (PI3K)-protein kinase B (Akt) signaling pathway (Fig. 1N). Fibroblasts and mural cells were divided into four cell clusters, including two fibroblast clusters (myofibroblast clusters: Fibroblasts_POSTN; inflammatory fibroblast clusters: Fibroblasts_CFD) and two mural cell clusters (MuralCells_ACTA2 and MuralCells_PDGFRB) using uniform manifold approximation and projection analysis (Fig. 1O and P). Compared with SPLC, DPLC-derived myofibroblasts had lower expression of some collagen proteins (COL1A1, COL1A2, COL3A1, COL6A2, COL6A3, and COL11A1), and such a decrease in cell activity was related to the reduction in collagen synthesis and secretion (Fig. S3A). Therefore, the decrease in expression of collagen-related genes may be the primary reason for the inhibition of bile acid metabolism and xenobiotic metabolism in DPLC (Fig. 1Q). Additionally, GSEA results showed that myofibroblasts were mainly involved in the epithelial–mesenchymal transition, TNFα signaling via NF-κB, inflammatory response, and interferon-gamma (IFN-γ) response (Fig. S3B). The KEGG analysis showed that the up-regulated differentially expressed genes were mainly involved in immune-related signaling pathways and endocrine-related signaling pathways (Table S3). Our study also found that the thyroid-stimulating hormone (TSH) levels in the blood of SPLC and SPTC were within the normal range (Fig. S1E). TSH levels in the DPLC group were significantly reduced, and the number of female patients was significantly higher than males (Fig. S1E and S1F). The data suggest that the level of thyroid hormones may be associated with the risk of DPLT. The levels of free triiodothyronine (FT3) and free thyroxine (FT4) in the DPLC group were higher than in the SPLC group (Fig. S1G and S1H). However, both values were within the normal range, indicating that DPLT patients belong to subclinical hyperthyroidism and have endocrine disorders. The above results reveal that the occurrence of double primary tumors is related to endocrine factors.

In conclusion, our study revealed that CD8Teff_GZMK cell subsets, Macrophages_CCL18 cell subsets, and myofibroblasts inhibited tumor activity in DPLC patients by anti-tumor immune response, reduction of pro-inflammatory cytokine expression, and inflammatory response. The differential genes in endothelial cells, epithelial cells, and fibroblasts of DPLC patients were mainly involved in endocrine-related pathways, among which the differential genes in valvular endothelial cell clusters and basal cell subsets were involved in the response to insulin and insulin-like growth factor by activating the PI3K-Akt-mTOR signaling pathway, thereby promoting malignant cell transformation in DPLC patients. These findings may improve the understanding of DPLT pathogenesis.

## CRediT authorship contribution statement

**Erteng Jia:** Writing – original draft, Funding acquisition, Data curation. **Lixia Zhang:** Data curation. **Yong Ge:** Data curation. **Li Li:** Data curation. **He Zhang:** Writing – review & editing. **Hao Zhang:** Conceptualization.

## Ethics declaration

This study was approved by the Ethics Committees of Affiliated Hospital of Xuzhou Medical University (No. XYFY2023-KL497-01). Informed consent was obtained from all the subjects involved in the study.

## Data availability

The raw data that support the findings of this study are available from the corresponding author upon reasonable request.

## Funding

This work was supported by funds from the National Natural Science Foundation of China (No.82472885 to Hao Zhang, 82500535 to Erteng Jia and 82300521 to He Zhang), the Noncommunicable Chronic Diseases-National Science and Technology Major Project (No. 2024ZD0529400 and 2024ZD0529405 to Hao Zhang), the Social Development Projects of Key R&D Programs in Xuzhou City (No. KC22097 to Hao Zhang), the XZHMU-QL Joint Research Fund (No. QL-YB018 to Erteng Jia), the Natural Science Foundation of the Jiangsu Higher Education Institutions of China (25KJD310003 to Erteng Jia), and the Xuzhou Medical University 2022 Scientific Research Launch Fund for Introducing High level Talents (No. D2022016 to Erteng Jia).

## Conflict of interests

The authors declared no competing interests.

## Figures and Tables

**Supplementary Figure S1:**
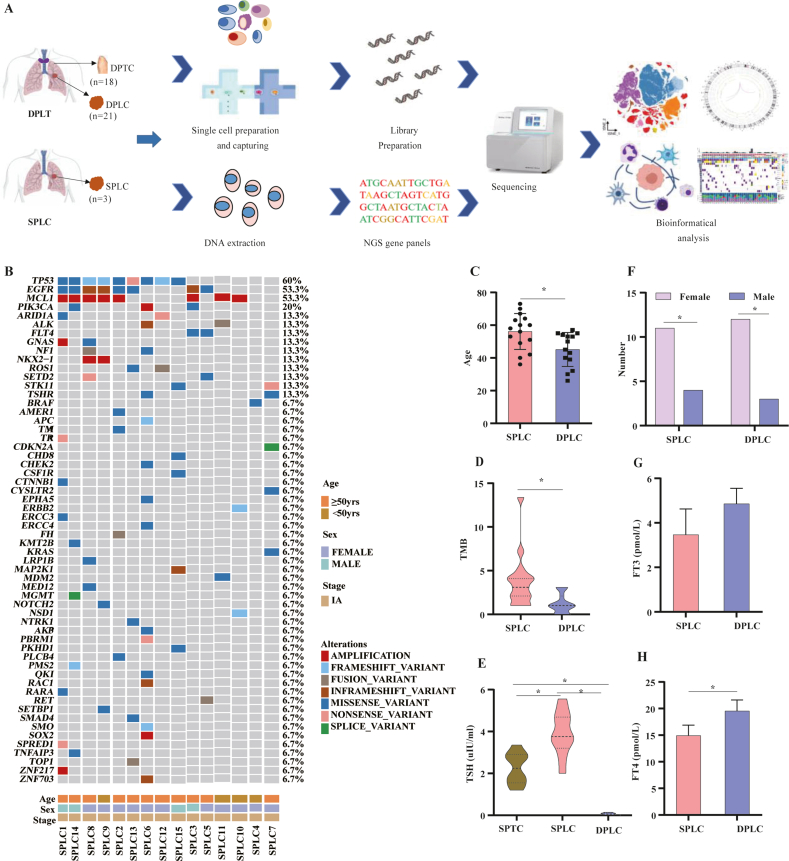
Clinical characteristics and large panel sequencing analysis. **(A)** Schematic diagram of the experimental workflow of this study. **(B)** Mutational profile of single primary lung cancer (SPLC) patients. **(C–H)** Analysis of clinical indicators of double primary lung cancer (DPLC) and SPLC patients, including (C) age, (D) tumor mutation burden, (E) thyroid-stimulating hormone (TSH), (F) sex, (G) free triiodothyronine (FT3), and (H) free thyroxine (FT4).

**Supplementary Figure S2:**
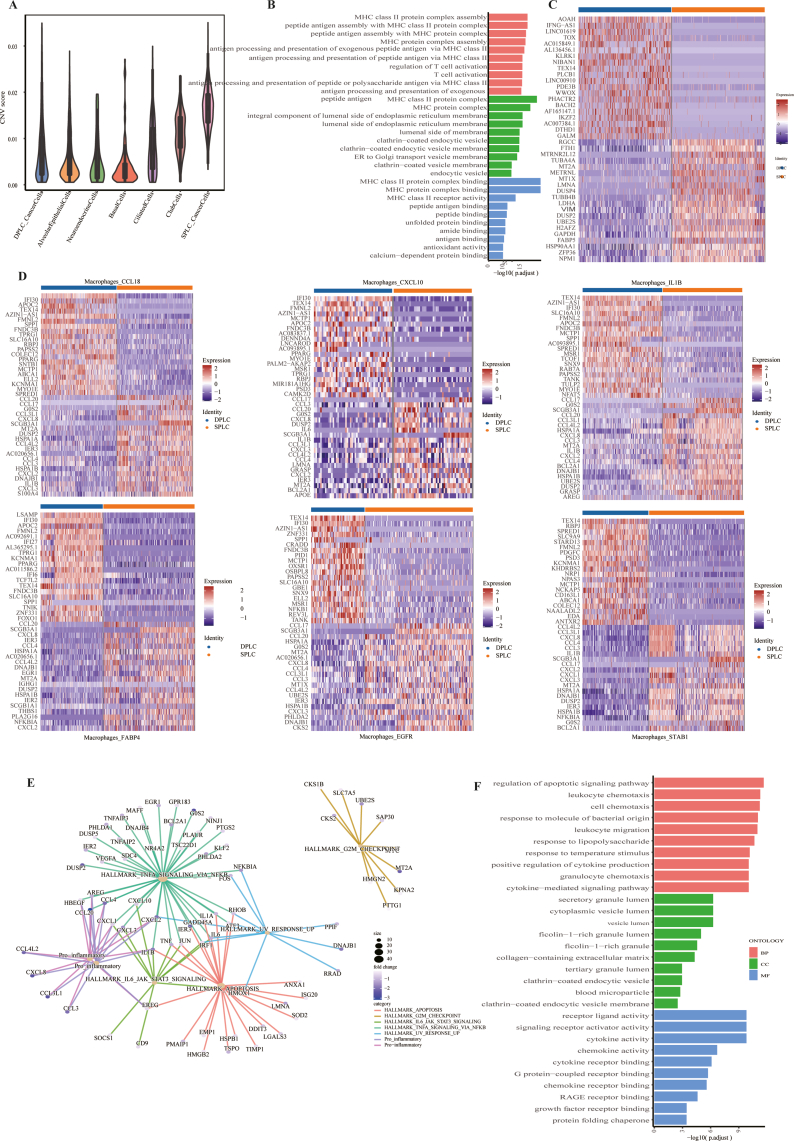
The functional role of macrophage cell subsets and T/natural killer cell subsets in double primary lung cancer (DPLC) and single primary lung cancer (SPLC). **(A)** Comparison of copy number variation in cancer cells between DPLC and SPLC patients. (B) Gene ontology (GO) enrichment analysis of up-regulated differentially expressed genes in CD8Teff_GZMK cell clusters. **(C)** Differential expression analysis of CD8Teff_GZMK cell subsets in different patients. **(D)** Differential expression analysis of different macrophage cell subsets in different patients. **(E, F)** Gene set enrichment analysis (GSEA) and GO analysis of down-regulated differentially expressed genes in macrophages of DPLC patients.

**Supplementary Figure S3:**
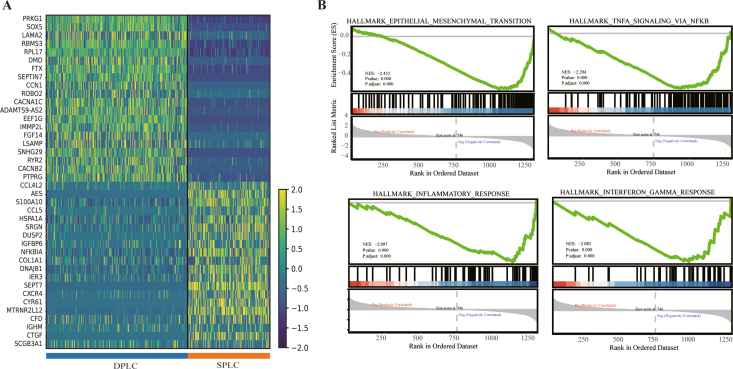
Fibroblasts and mural cell clusters. **(A)** Differential expression analysis of fibroblasts in different patients. **(B)** Gene set enrichment analysis (GSEA) revealed the biological functions involved in different cell subsets.
