# Supplementary material for: Single-cell RNA sequencing and large-panel NGS analysis reveal transcriptional heterogeneity and genomic characteristics of double primary lung cancer and thyroid cancer
Source: Genes Dis. 2025 Oct 22;13(4):101889. doi: 10.1016/j.gendis.2025.101889 (PMC12995690; doi:10.1016/j.gendis.2025.101889)
Supplement: Multimedia component 1 [file mmc1.docx]

**Single‐cell RNA-seq and large-panel NGS analysis reveals transcriptional heterogeneity and genomic characteristics of double primary lung cancer and thyroid cancer**

**Material and Methods**

**Clinical Sample collection and preparation**

All specimens were collected following the guidelines of the U.S. Health Insurance Portability and Accountability Act (HIPAA) of 1996 and supervised by the ethics committee of the hospital. All patients were diagnosed with primary tumours and were not treated. During the surgery, three lung cancer tissues (DPLC) from double primary lung cancer and thyroid cancer (DPLT) patients were obtained for single-cell sequencing. Additionally, three single primary lung cancer tissues (SPLC) were collected as controls. The clinical characteristics, including patient gender, age of cancer diagnosis, and pathological subtype, are given in Supplementary Table S1.

Retrospectively, the formalin-fixed paraffin-embedded (FFPE) tumour samples from 18 DPLT patients and 15 SPLC were screened at Affiliated Hospital of Xuzhou Medical University between May 2020 and June 2022. Information about patients’ gender, their age at cancer diagnosis, and pathological staging are shown in Table S2. Among these 33 tumour patients, 18 DPLC and 15 SPLC samples were available. Targeted sequencing of 437 cancer-related genes was performed in all samples. This study has been approved by the ethics committees of Affiliated Hospital of Xuzhou Medical University (XYFY2023-KL497-01).

**Single-cell RNA sequencing**

Lung cancer tissues were collected and immediately placed in SCelLiVe^®^, and the tissue was rapidly digested to prepare single-cell suspensions. According to the manufacturer’s instructions, construction of a cDNA library was undertaken using a 10x genomics Chromium Single Cell 3′ Library Kit. The libraries were sequenced using the Illumina Hiseq X Ten platform.

**DNA isolation and targeted DNA sequencing**

According to the manufacturer’s protocol, the genomic DNA from 18 DPLC FFPE tissues and 15 SPLC FFPE tissues were extracted using a QIAamp DNA FFPE Tissue Kit. A Qubit 3.0 fluorometer and Agilent instrument were used to evaluate the concentration and quality of extracted DNA. The total amount of DNA used for library construction was not less than 200 ng. Prior to library preparation, the genomic DNA was fragmented, and DNA fragments of 200-300 bp in size were screened. The screened DNA fragment was then end-repaired, and the A base and the adaptor sequence were added. Finally, the DNA product with the adaptor was purified, and the total library was increased by PCR amplification. In this study, DNA probe libraries hybridised with tumour driver genes were designed to enrich exon regions corresponding to target genes in high-throughput sequencing libraries using synthetic probe libraries. Presently, the 437-panel probe of Nanjing Shihe Gene Biotechnology Co., Ltd. is used as the tumour-related gene capture method. After DNA library enrichment, high-throughput sequencing was performed using the Illumina Hiseq sequencing platform PE150 kit. The average sequencing depth of tissue samples was not less than 1000 X.

**Pre-processing of scRNA-seq data**

Cell Ranger software (version 4.0.0, 10x Genomics) was used for sequencing data splitting and cell barcoding of raw single-cell RNA sequencing data. The raw sequencing data was then compared to the GRCh38 human reference genome to generate the original gene-cell expression matrix. In this study, low-quality cells were removed according to the following criteria: (1) the total number of detected genes was less than 200; (2) UMI below 800; (3) cells with more than 20% total mitochondrial genes. The gene expression matrix data was standardised using the “NormalizeData” function in the “Seurat” R package to generate a new gene expression matrix for subsequent downstream data analysis. The ‘FindClusters’ function in Seurat was used for unsupervised dimensionality reduction and clustering analysis of gene expression matrices, and the expression of canonical marker genes annotated the clusters. The inferCNV R package was used to distinguish DPLC and SPLC groups of malignant cells by inferring chromosomal CNVs based on the gene expression data. The AlveolarEpithelialCells, Ciliated cells, Club cells, Neuroendocrine cells, and Basal cells as normal reference cells were used to estimate CNVs for DPLC and SPLC groups’ potential tumour cell population.

**DEGs and Functional Enrichment Analysis**

The “FindAll-Markers” function in Seurat was used to analyse differentially expressed genes (DEGs), and used | FC |>2 and adjusted *p* value<0.05 as the threshold. The clusterProfiler package was used for GO, KEGG, and GSEA functional analysis of DEGs. We use the R package “GSVA” to score gene sets of biological processes for each sample.

**SNV/indel/CNV detection**

The quality control analysis of the raw data was carried out using Trimmomatic. The clean data was then aligned to the human reference genome using the BWA-MEM algorithm and default parameters to generate a SAM file. SAM files were converted into BAM files using Picard (1.119) and then filtered based on chromosome coordinates. VarScan 2 was used to identify single nucleotide variants (SNVs) and short insertion/deletion (indel), where the minimum variant allele frequency threshold was set to 0.01. The p-value threshold for identifying variants was set to 0.05 to generate variant identification format (VCF) files. ANNOVAR was used to annotate VCF results, annotate dbSNP IDs, assess clinical significance, and predict protein function effects using SIFT and PolyPhen software. Copy number variants (CNVs) were analysed with CNVkit. Finally, the tumour mutation burden (TMB) was analysed.

**Statistical Analysis**

All statistical analyses were analysed using the SPSS 22.0 software. Tukey’s test was used to analyse the data for significant differences between the two groups. Differences were considered significant at *P* < 0.05.
