# Supplementary material for: Single-cell RNA sequencing and large-panel NGS analysis reveal transcriptional heterogeneity and genomic characteristics of double primary lung cancer and thyroid cancer
Source: Genes Dis. 2025 Oct 22;13(4):101889. doi: 10.1016/j.gendis.2025.101889 (PMC12995690; doi:10.1016/j.gendis.2025.101889)
Supplement: Multimedia component 2 [file mmc2.docx]

Table S1. The gene mutation characteristics of the DPLC patients and SPLC patients.

| Sample | Type | Gene | AAChange | ExonicFunc | CopyNumber |
| --- | --- | --- | --- | --- | --- |
| DPLC1 | Mutant | CYP2D6 | c.953T>A(p.L318H) | missense_variant |  |
| DPLC1 | Mutant | KMT2C | c.1121C>A(p.A374E) | missense_variant |  |
| DPLC1 | Mutant | KRAS | c.34G>T(p.G12C) | missense_variant |  |
| DPLC1 | CNV | CDK4 | . | . | 5.8872 |
| DPLC1 | CNV | MCL1 | . | . | 8.8434 |
| DPLC2 | Mutant | MAP2K1 | c.304_309del(p.E102_I103del) | inframe_deletion |  |
| DPLC3 | Mutant | ZNF703 | c.1579G>T(p.G527C) | missense_variant | . |
| DPLC3 | Mutant | EGFR | c.2573T>G(p.L858R) | missense_variant | . |
| DPLC3 | CNV | CDK4 | . | . | 8.9756 |
| DPLC3 | CNV | MDM2 | . | . | 21.5394 |
| DPLC4 | Germline | MPL | c.413del(p.I138Tfs*28) | frameshift_variant | . |
| DPLC4 | Mutant | ERBB2 | c.2327delinsTTGT(p.G776delinsVV) | inframe_insertion | . |
| DPLC5 | Mutant | ERBB2 | c.2313_2324dup(p.Y772_A775dup) | inframe_insertion | . |
| DPLC6 | Mutant | AXIN2 | c.1253C>T(p.P418L) | missense_variant | . |
| DPLC6 | Mutant | BRAF | c.1803A>T(p.K601N) | missense_variant | . |
| DPLC8 | Mutant | ERBB2 | c.2313_2324dup(p.Y772_A775dup) | inframe_insertion | . |
| DPLC8 | Mutant | SMARCA4 | c.2059A>G(p.K687E) | missense_variant | . |
| DPLC8 | CNV | EPCAM | . | . | 0.9246 |
| DPLC9 | Mutant | POT1 | c.473A>T(p.K158I) | missense_variant | . |
| DPLC10 | Mutant | ERBB3 | c.1150C>T(p.L384F) | missense_variant | . |
| DPLC10 | Mutant | EGFR | c.2573T>G(p.L858R) | missense_variant | . |
| DPLC10 | CNV | CDK4 | . | . | 4.4828 |
| DPLC10 | CNV | MCL1 | . | . | 6.3304 |
| DPLC11 | Mutant | EGFR | c.2573T>G(p.L858R) | missense_variant | . |
| DPLC11 | CNV | MCL1 | . | . | 6.3112 |
| DPLC12 | Mutant | NSD1 | c.518G>A(p.C173Y) | missense_variant | . |
| DPLC12 | Mutant | PALLD | c.3180C>G(p.H1060Q) | missense_variant | . |
| DPLC12 | Mutant | EGFR | c.2239_2259delinsAAT  (p.L747_P753delinsN) | inframe_deletion | . |
| DPLC12 | Mutant | FBXW7 | c.1498C>T(p.Q500*) | stop_gained | . |
| DPLC12 | Mutant | ERCC2 | c.949G>A(p.E317K) | missense_variant&splice_region_variant | . |
| DPLC12 | Mutant | FLT3 | c.2617T>A(p.S873T) | missense_variant | . |
| DPLC12 | Mutant | APC | c.2828_2853del(p.S943Cfs*11) | frameshift_variant | . |
| DPLC12 | Mutant | CTNNB1 | c.121A>G(p.T41A) | missense_variant | . |
| DPLC12 | Mutant | APC | c.3927_3931del(p.E1309Dfs*4) | frameshift_variant | . |
| DPLC12 | Mutant | CREBBP | c.4322G>A(p.R1441Q) | missense_variant | . |
| DPLC12 | Mutant | APC | c.4682_4684delinsGA(p.K1561Rfs*4) | frameshift_variant | . |
| DPLC13 | Mutant | ERBB2 | c.2313_2324dup(p.Y772_A775dup) | inframe_insertion | . |
| DPLC13 | Mutant | ERBB4 | c.217G>A(p.D73N) | missense_variant | . |
| DPLC14 | Mutant | KRAS | c.35G>T(p.G12V) | missense_variant | . |
| DPLC14 | CNV | MCL1 | . | . | 4.415 |
| DPLC15 | Mutant | EGFR | c.2237_2254del(p.E746_S752delinsA) | inframe_deletion | . |
| DPLC16 | Mutant | MAP2K1 | c.303_308del(p.E102_I103del) | inframe_deletion | . |
| DPLC16 | Mutant | FANCF | c.157C>T(p.P53S) | missense_variant | . |
| DPLC16 | CNV | MCL1 | . | . | 4.5404 |
| DPLC18 | Mutant | BARD1 | c.1444C>T(p.Q482*) | stop_gained | . |
| DPLC18 | Mutant | APC | c.7766A>T(p.E2589V) | missense_variant | . |
| DPLC18 | Mutant | RAD51 | c.751A>C(p.M251L) | missense_variant | . |
| DPLC18 | Mutant | EGFR | c.2573T>G(p.L858R) | missense_variant | . |
| DPLC18 | Mutant | CSF1R | c.1646G>A(p.R549H) | missense_variant | . |
| DPLC18 | Mutant | EPHA2 | c.262C>T(p.R88*) | stop_gained | . |
| DPLC18 | CNV | MCL1 | . | . | 8.2072 |
| SPLC1 | Mutant | EGFR | c.2573T>G(p.L858R) | missense_variant |  |
| SPLC1 | Mutant | ATR | c.6530G>A(p.W2177*) | nonsense variant |  |
| SPLC1 | Mutant | TP53 | c.398T>A(p.M133K) | missense_variant |  |
| SPLC1 | Mutant | CTNNB1 | c.110C>T(p.S37F) | missense_variant |  |
| SPLC1 | Mutant | CTNNB1 | c.98C>T(p.S33F) | missense_variant |  |
| SPLC1 | Mutant | EGFR | c.2497T>G(p.L833V) | missense_variant |  |
| SPLC1 | CNV | GNAS |  |  | 5.54 |
| SPLC1 | CNV | MCL1 |  |  | 6.12 |
| SPLC1 | Mutant | SPRED1 | c.1142C>G(p.S381*) | nonsense variant |  |
| SPLC1 | CNV | ZNF217 | - |  |  |
| SPLC1 | Mutant | ARID1A |  | nonsense variant |  |
| SPLC1 | Mutant | ARID1A | ARID1A:exon17~AGBL4:exon4 | fusion variant |  |
| SPLC1 | Mutant | ERCC3 | c.2086A>T(p.M696L) | missense_variant |  |
| SPLC1 | Mutant | RARA | Missense mutation in exon 6 of pD221V | missense_variant |  |
| SPLC2 | Mutant | EGFR | c.2573T>G(p.L858R) | missense_variant |  |
| SPLC2 | Mutant | TP53 | c.374C>A(p.T125K) | missense_variant |  |
| SPLC2 | CNV | MCL1 |  |  | 4.54 |
| SPLC2 | Mutant | AMER1 |  | missense_variant |  |
| SPLC2 | Mutant | ATM | c.8264A>T(p.Y2755F) | missense_variant |  |
| SPLC2 | Mutant | FH | FH:exon5~IGR (downstream BRINP3) | fusion variant |  |
| SPLC2 | Mutant | PLCB4 | c.803C>T(p.A268V) | missense_variant |  |
| SPLC3 | Mutant | EGFR | c.2300_2308dup(p.A767_V769dup) | frameshift variant |  |
| SPLC3 | Mutant | PIK3CA | c.1633G>A(p.E545K) | missense_variant |  |
| SPLC3 | CNV | MCL1 |  |  | 4.7 |
| SPLC3 | Mutant | FLT4 | c.3092G>A(p.R1031Q) | missense_variant |  |
| SPLC4 | Mutant | BRAF | c.1799T>A(p.V600E) | missense_variant |  |
| SPLC4 | CNV | BRAF |  |  | 6.5 |
| SPLC5 | Mutant | EGFR | c.2573T>G(p.L858R) | missense_variant |  |
| SPLC5 | Mutant | RET | RET:exon9~MICU1:exon12 |  |  |
| SPLC5 | Mutant | FLT4 | c.3205G>A (p.V1069I) | missense_variant |  |
| SPLC5 | Mutant | SETD2 | c.485C>T(p.A162V) | missense_variant |  |
| SPLC6 | Mutant | TP53 |  | missense_variant |  |
| SPLC6 | Mutant | APC | c.4648_4651del(p.E1550Kfs*14) | frameshift variant |  |
| SPLC6 | Mutant | PBRM1 | c.1028C>G(p.S343*) | nonsense variant |  |
| SPLC6 | CNV | PIK3CA | - |  | 4.8 |
| SPLC6 | CNV | SOX2 |  |  | 9.3 |
| SPLC6 | Mutant | ALK | c.4554_4556del(p.K1518del) | frameshift variant |  |
| SPLC6 | Mutant | CHEK2 | c.668G>A(p.R223H) | missense_variant |  |
| SPLC6 | Mutant | EPHA5 | c.495C>A(p.N165K) | missense_variant |  |
| SPLC6 | Mutant | ERCC4 | c.2445G>T(p.E815D) | missense_variant |  |
| SPLC6 | Mutant | NF1 | c.6133A>T(p.T2045S) | missense_variant |  |
| SPLC6 | Mutant | PAK3 | c.712G>C(p.D238H) | missense_variant |  |
| SPLC6 | Mutant | QKI | c.283G>T(p.D95Y) | missense_variant |  |
| SPLC6 | Mutant | RAC1 | c.496_498del(p.K166del) | frameshift variant |  |
| SPLC6 | Mutant | SMO | c.1278_1279del(p.F427Lfs*12) | frameshift variant |  |
| SPLC6 | Mutant | TSHR | c.572C>T(p.S191L) | missense_variant |  |
| SPLC6 | Mutant | ZNF703 | c.1537_1542del(p.A513_A514del) | frameshift variant |  |
| SPLC7 | Mutant | CDKN2A | c.150+1G>C | splicing variant |  |
| SPLC7 | Mutant | KRAS | c.35G>A(p.G12D) | missense_variant |  |
| SPLC7 | Mutant | STK11 | c.784A>T(p.K262*) | nonsense variant |  |
| SPLC7 | Mutant | CYSLTR2 | c.62G>A(p.G21D) | missense_variant |  |
| SPLC7 | Mutant | TSHR | c.1169G>A(p.C390Y) | missense_variant |  |
| SPLC8 | Mutant | EGFR | c.2235_2249del(p.E746_A750del) | frameshift variant |  |
| SPLC8 | Mutant | TP53 | c.78del(p.P27Lfs*17) | frameshift variant |  |
| SPLC8 | CNV | MCL1 | - |  | 4.63 |
| SPLC8 | CNV | NKX2-1 |  |  | 5.63 |
| SPLC8 | Mutant | SETD2 | c.3615T>G(p.Y1205*) | nonsense variant |  |
| SPLC8 | Mutant | GNAS |  | missense_variant |  |
| SPLC8 | Mutant | LRP1B | c.8398G>A(p.A2800T) | missense_variant |  |
| SPLC8 | Mutant | MED12 | c.2258G>A(p.R753Q) | missense_variant |  |
| SPLC8 | Mutant | NF1 | NF1:exon35~FRMD6:5'UTR | fusion variant |  |
| SPLC8 | Mutant | NF1 | GNG2:5'UTR~NF1:exon7 | fusion variant |  |
| SPLC9 | Mutant | EGFR | c.2235_2249del(p.E746_A750del) | frameshift variant |  |
| SPLC9 | Mutant | TP53 | c.403del(p.c135Afs*35) | frameshift variant |  |
| SPLC9 | CNV | MCL1 |  |  | 6.63 |
| SPLC9 | CNV | NKX2-1 |  |  | 8.25 |
| SPLC9 | Mutant | NOTCH2 | c.6575C>G(p.P2192R) | missense_variant |  |
| SPLC9 | Mutant | SETBP1 | c.1237C>A(p.P413T) | missense_variant |  |
| SPLC10 | Mutant | ERBB2 | c.2313_2324dup(p.Y772_A775dup) | frameshift variant |  |
| SPLC10 | CNV | MCL1 | - |  | 4.33 |
| SPLC10 | Mutant | NSD1 | c.6397_6407del(p.T2133Afs*3) | frameshift variant |  |
| SPLC11 | Mutant | ALK | EML4:exon18~ALK:exon20 | fusion variant |  |
| SPLC11 | Mutant | ALK | ALK:exon20~CREBL2:exon4 | fusion variant |  |
| SPLC11 | CNV | MCL1 |  |  | 4.89 |
| SPLC11 | Mutant | MDM2 | c.964C>T(p.P322S) | missense_variant |  |
| SPLC12 | Mutant | ROS1 | SDC4:exon4~ROS1:exon32 | fusion variant |  |
| SPLC12 | Mutant | ARID1A | c.2404C>T(p.Q802*) | nonsense variant |  |
| SPLC12 | Mutant | TP53 | c.277_278del(p.L93Vfs*55) | frameshift variant |  |
| SPLC13 | Mutant | EGFR | c.2573T>G(p.L858R) | missense_variant |  |
| SPLC13 | Mutant | TP53 | c.892G>T(p.E298*) | nonsense variant |  |
| SPLC13 | Mutant | SMAD4 | c.1081C>T(p.R361C) | missense_variant |  |
| SPLC13 | Mutant | NTRK1 | c.1358C>T(p.P453L) | missense_variant |  |
| SPLC13 | Mutant | ROS1 | c.5679G>T(p.K1893N) | missense_variant |  |
| SPLC13 | Mutant | TOP1 | IGR (upstream GALNT17)~TOP1:exon2 | fusion variant |  |
| SPLC14 | Mutant | EGFR | c.2573T>G(p.L858R) | missense_variant |  |
| SPLC14 | Mutant | PIK3CA | c.3140A>G(p.H1047R) | missense_variant |  |
| SPLC14 | CNV | MCL1 |  |  | 4.45 |
| SPLC14 | Mutant | MGMT | C.275-1G>A | splicing variant |  |
| SPLC14 | Mutant | PMS2 | c.776_786del(p.C259Sfs*3) | frameshift variant |  |
| SPLC14 | Mutant | EGFR | c.2159C>T(p.S720F) | missense_variant |  |
| SPLC14 | Mutant | KMT2B | c.5176G>A(p.D1726N) | missense_variant |  |
| SPLC14 | Mutant | TNFAIP3 | c.1433G>T(p.C478F) | missense_variant |  |
| SPLC14 | Mutant | TP53 | c.794T>G(p.L265R) | missense_variant |  |
| SPLC15 | Mutant | STK11 | c.647C>T(p.S216F) | missense_variant |  |
| SPLC15 | Mutant | TP53 | c.577C>T(p.H193Y) | missense_variant |  |
| SPLC15 | Mutant | MAP2K1 | c.303_309delinsA(p.E102_1103del) | frameshift variant |  |
| SPLC15 | Mutant | CHD8 | c.5602G>A(p.E1868K) | missense_variant |  |
| SPLC15 | Mutant | CSF1R | c.1577T>C(p.M526T) | missense_variant |  |
| SPLC15 | Mutant | PKHD1 | c.5402G>T(p.G1801V) | missense_variant |  |
