# Supplementary material for: Single-cell RNA sequencing and large-panel NGS analysis reveal transcriptional heterogeneity and genomic characteristics of double primary lung cancer and thyroid cancer
Source: Genes Dis. 2025 Oct 22;13(4):101889. doi: 10.1016/j.gendis.2025.101889 (PMC12995690; doi:10.1016/j.gendis.2025.101889)
Supplement: Multimedia component 4 [file mmc4.docx]

Table S3. Functional enrichment analysis of upregulated differentially expressed genes in endothelial cells, epithelial cells, and fibroblasts of patients with DPLC and SPLC.

| ID | Description | pvalue | p.adjust | qvalue |
| --- | --- | --- | --- | --- |
| hsa04120 | Ubiquitin mediated proteolysis | 3.34E-12 | 6.52E-10 | 4.82E-10 |
| hsa04520 | Adherens junction | 1.62E-10 | 1.58E-08 | 1.17E-08 |
| hsa04510 | Focal adhesion | 2.60E-09 | 1.69E-07 | 1.25E-07 |
| hsa05200 | Pathways in cancer | 1.59E-08 | 7.75E-07 | 5.73E-07 |
| hsa04360 | Axon guidance | 3.09E-08 | 1.21E-06 | 8.92E-07 |
| hsa04310 | Wnt signaling pathway | 3.88E-08 | 1.26E-06 | 9.32E-07 |
| hsa05220 | Chronic myeloid leukemia | 1.39E-06 | 3.46E-05 | 2.56E-05 |
| hsa04810 | Regulation of actin cytoskeleton | 1.42E-06 | 3.46E-05 | 2.56E-05 |
| hsa05215 | Prostate cancer | 2.28E-06 | 4.94E-05 | 3.65E-05 |
| hsa04662 | B cell receptor signaling pathway | 8.69E-06 | 0.000169 | 0.000125 |
| hsa05221 | Acute myeloid leukemia | 1.41E-05 | 0.000241 | 0.000178 |
| hsa04012 | ErbB signaling pathway | 1.48E-05 | 0.000241 | 0.000178 |
| hsa04010 | MAPK signaling pathway | 1.75E-05 | 0.000256 | 0.000189 |
| hsa04144 | Endocytosis | 1.83E-05 | 0.000256 | 0.000189 |
| hsa04720 | Long-term potentiation | 2.82E-05 | 0.000324 | 0.000239 |
| hsa05211 | Renal cell carcinoma | 2.82E-05 | 0.000324 | 0.000239 |
| hsa05212 | Pancreatic cancer | 2.82E-05 | 0.000324 | 0.000239 |
| hsa05213 | Endometrial cancer | 3.55E-05 | 0.000385 | 0.000285 |
| hsa05210 | Colorectal cancer | 4.27E-05 | 0.000438 | 0.000324 |
| hsa04660 | T cell receptor signaling pathway | 4.58E-05 | 0.000447 | 0.000331 |
| hsa05222 | Small cell lung cancer | 8.68E-05 | 0.000806 | 0.000596 |
| hsa04530 | Tight junction | 0.000187 | 0.001662 | 0.001229 |
| hsa04062 | Chemokine signaling pathway | 0.000286 | 0.002429 | 0.001796 |
| hsa04722 | Neurotrophin signaling pathway | 0.000414 | 0.003367 | 0.00249 |
| hsa04070 | Phosphatidylinositol signaling system | 0.000484 | 0.003773 | 0.00279 |
| hsa04666 | Fc gamma R-mediated phagocytosis | 0.000549 | 0.004116 | 0.003044 |
| hsa04270 | Vascular smooth muscle contraction | 0.00101 | 0.007291 | 0.005392 |
| hsa04670 | Leukocyte transendothelial migration | 0.001159 | 0.008072 | 0.00597 |
| hsa04710 | Circadian rhythm - mammal | 0.00123 | 0.008268 | 0.006115 |
| hsa04540 | Gap junction | 0.001472 | 0.009571 | 0.007078 |
| hsa04350 | TGF-beta signaling pathway | 0.001622 | 0.010202 | 0.007545 |
| hsa04912 | GnRH signaling pathway | 0.003138 | 0.01912 | 0.01414 |
| hsa05131 | Shigellosis | 0.003531 | 0.020868 | 0.015432 |
| hsa00562 | Inositol phosphate metabolism | 0.003819 | 0.021902 | 0.016198 |
| hsa04150 | mTOR signaling pathway | 0.004095 | 0.022816 | 0.016874 |
| hsa05214 | Glioma | 0.005823 | 0.031539 | 0.023325 |
| hsa05100 | Bacterial invasion of epithelial cells | 0.006132 | 0.032318 | 0.0239 |
| hsa04916 | Melanogenesis | 0.006695 | 0.034355 | 0.025407 |
| hsa04664 | Fc epsilon RI signaling pathway | 0.008403 | 0.042016 | 0.031072 |
| hsa04320 | Dorso-ventral axis formation | 0.009425 | 0.044221 | 0.032703 |
| hsa05160 | Hepatitis C | 0.009487 | 0.044221 | 0.032703 |
| hsa00512 | Mucin type O-Glycan biosynthesis | 0.009525 | 0.044221 | 0.032703 |
| hsa04914 | Progesterone-mediated oocyte maturation | 0.010965 | 0.049724 | 0.036773 |
