# Supplementary material for: Single-cell RNA sequencing and large-panel NGS analysis reveal transcriptional heterogeneity and genomic characteristics of double primary lung cancer and thyroid cancer
Source: Genes Dis. 2025 Oct 22;13(4):101889. doi: 10.1016/j.gendis.2025.101889 (PMC12995690; doi:10.1016/j.gendis.2025.101889)
Supplement: Multimedia component 5 [file mmc5.docx]

Table S4. Functional enrichment analysis of downregulated differentially expressed genes in endothelial cells, epithelial cells, and fibroblasts of patients with DPLC and SPLC.

| ID | Description | pvalue | p.adjust | qvalue |
| --- | --- | --- | --- | --- |
| hsa03010 | Ribosome | 1.29E-40 | 2.23E-38 | 1.94E-38 |
| hsa03040 | Spliceosome | 9.43E-08 | 8.16E-06 | 7.1E-06 |
| hsa04621 | NOD-like receptor signaling pathway | 3.24E-05 | 0.001868 | 0.001625 |
| hsa05323 | Rheumatoid arthritis | 9.02E-05 | 0.003902 | 0.003395 |
| hsa04612 | Antigen processing and presentation | 0.000175 | 0.006055 | 0.005268 |
| hsa05142 | Chagas disease (American trypanosomiasis) | 0.000393 | 0.011338 | 0.009865 |
| hsa04062 | Chemokine signaling pathway | 0.000797 | 0.017373 | 0.015116 |
| hsa05332 | Graft-versus-host disease | 0.000803 | 0.017373 | 0.015116 |
| hsa03050 | Proteasome | 0.001136 | 0.019647 | 0.017095 |
| hsa04940 | Type I diabetes mellitus | 0.001136 | 0.019647 | 0.017095 |
| hsa05012 | Parkinson's disease | 0.001735 | 0.025861 | 0.022502 |
| hsa05330 | Allograft rejection | 0.001794 | 0.025861 | 0.022502 |
| hsa04640 | Hematopoietic cell lineage | 0.00199 | 0.026477 | 0.023038 |
| hsa05016 | Huntington's disease | 0.00278 | 0.034354 | 0.029892 |
| hsa05416 | Viral myocarditis | 0.003316 | 0.038247 | 0.033279 |
| hsa05140 | Leishmaniasis | 0.003702 | 0.040023 | 0.034824 |
| hsa04141 | Protein processing in endoplasmic reticulum | 0.005108 | 0.049449 | 0.043025 |
| hsa04145 | Phagosome | 0.005145 | 0.049449 | 0.043025 |
| hsa04623 | Cytosolic DNA-sensing pathway | 0.005438 | 0.049511 | 0.043079 |
